# Prevalence and progress of underdiagnosis of probable dementia: a repeated cross-sectional study in 19 European countries

**DOI:** 10.1186/s12916-025-04196-7

**Published:** 2025-07-01

**Authors:** Jiazhou Yu, Pengyun Wang, Shangkun Xie, Jay Amin, Christoph Mueller, Xiaohui Hou, Xi Chen, Benjamin R. Underwood, Shangfeng Tang, Shanquan Chen

**Affiliations:** 1 Department of Medicine and Therapeutics, The Chinese University of Hong Kong, Hong Kong SAR, China; 2 University of Oxford, Oxford, UK; 3 School of Economics, Nankai University, Tianjin, China; 4 Clinical Neurosciences, Faculty of Medicine, University of Southampton, Southampton, UK; 5 Memory Assessment and Research Centre, Southern Health NHS Foundation Trust, Southampton, UK; 6 King’s College London, London, UK; 7 South London and Maudsley NHS Foundation Trust, London, UK; 8 The World Bank, Washington, DC 20433 USA; 9 School of Public Health, Yale University, New Haven, CT USA; 10 Department of Psychiatry, University of Cambridge, Cambridge, UK; 11 Cambridgeshire and Peterborough NHS Foundation Trust, Cambridgeshire, UK; 12 School of Medicine and Health Management, Tongji Medical College of Huazhong University of Science and Technology, Qiaokou District, No.13, Hangkong Road, Wuhan, 430030 Hubei China; 13 School of Public Health, LKS Faculty of Medicine, the University of Hong Kong, Hong Kong SAR, China; 14 International Centre for Evidence in Disability, London School of Hygiene & Tropical Medicine, London, WC1E 7HT UK

**Keywords:** Dementia, Diagnosis, Undiagnosed, Disparity, European

## Abstract

**Background:**

Underdiagnosis of dementia remains a significant public health challenge in Europe, with nearly half of those meeting clinical criteria not receiving a formal diagnosis. Recent healthcare initiatives have aimed to improve diagnostic processes, but the extent of progress varies across countries.

**Methods:**

We analyzed data of 10,402 participants from the Survey of Health, Ageing, and Retirement in Europe (SHARE) across 19 countries from 2011–2015 to 2015–2019. Underdiagnosis of probable dementia was defined as probable dementia (based on cognition score) without a confirmed diagnosis. Weighted logistic regression was conducted to examine temporal trends in underdiagnosis of probable dementia and to identify individual- and country-level predictors of progress in diagnosis.

**Results:**

A significant reduction in underdiagnosis of dementia was observed between the two periods, consistent across most countries. Progress in diagnosis was modified by country-level factors such as number of psychiatrists, formal long-term care worker at home or institutions, and positron emission tomography and individual-level factors including age, education, retirement status, nursing home residency, multimorbidity, and healthcare utilization patterns.

**Conclusions:**

The decreasing trend in underdiagnosis highlights the importance of targeted interventions including investment in psychiatric care services. Understanding remaining disparities is crucial for informing health policies and addressing inequalities to dementia diagnosis and care.

## Background

In Europe, dementia constitutes a substantial public health challenge, primarily due to its prevalence and associated socioeconomic impact [1, 2]. As of 2010, approximately10 million individuals were diagnosed with dementia in the European Union, a number anticipated to double by 2050 in line with aging trends [2, 3]. Characterized by a progressive deterioration in cognitive function, dementia can significantly affect older individuals in various aspects. The condition not only impairs patients’ cognitive and physical functions but also substantially affects the emotional, psychological, and social well-being of both affected individuals and their families [4, 5]. Furthermore, dementia presents a considerable economic burden, with direct annual costs per person ranging from EUR 253 to EUR 859 across European countries [1].

The World Health Organization’s (WHO) Global Action Plan on the Public Health Responses to Dementia 2017–2025 emphasized the critical importance of early detection in dementia for effective disease management [6]. Early diagnosis enables timely intervention strategies and facilitates comprehensive and informed communication among medical practitioners, patients, and their caregivers [6, 7]. Specifically, the WHO has established a goal of “in at least 50% of countries, as a minimum, 50% of the estimated number of people with dementia are diagnosed by 2025” [6]. A systematic review of studies predominantly published before 2013 revealed common underdiagnosis of dementia across Europe showing that only about half of individuals (43.1% in the UK and 58.2% in other European countries) meeting the clinical criteria for dementia had received an official diagnosis [8]. The rate of underdiagnosis varied considerably by region, ranging from 31.0% in the Netherlands to 70.0% in Spain [8]. As the target year of 2025 approaches, it is important to update these estimations to assess progress and identify any areas for improvement.

Previous studies have tempted to identify potential predictors of the underdiagnosis of dementia, but the findings remained inconsistent. For example, lower socioeconomic status was identified as a significant contributor to underdiagnosis in China [9] and the UK [10], but not in Japan [11]. Advanced age was associated with increased underdiagnosis in the UK [12] and US [13], but a meta-analysis synthesizing data across multiple countries reported contrary results [8]. Social support predicted reduced dementia underdiagnosis in the UK [12] and Canada [14] but predicted increased underdiagnosis in China [9], and the association was non-significant in Japan [11]. The regional disparity in these associations and the prevalence of underdiagnosed suggests the potential effects of regional healthcare policies and social norms [8]. However, the existing evidence on underdiagnosis rates of dementia predominately comes from surveys independently collected in each country with varying methodologies and timeframes, which limits standardized cross-regional comparisons and the identification of contributing factors of the regional differences. Adopting a longitudinal and multi-national design allows for examination of progress in dementia diagnosis and its predictors, which is essential for developing effective interventions and addressing cross-regional disparities.

This study aims to evaluate the temporal trends in the underdiagnosis of dementia across 19 European countries, by age, sex, and socioeconomic status. The study also examines the individual-level and country-level factors associated with variations in the rate of dementia underdiagnosis, which may explain the temporal shifts over time.

## Methods

### Study design and data source

We utilized publicly available datasets from the Survey of Health, Ageing, and Retirement in Europe (SHARE), a European version of Health and Retirement Survey (HRS) covering 19 countries [15–18]. Details of study design, data collection, sampling methodologies, and quality control procedures are described elsewhere [19, 20]. In brief, SHARE is a biennial, multi-national survey targeting individuals aged 50 and above, employing centrally standardized methods across participating nations to facilitate cross-country comparisons. Participants in SHARE were recruited through probability-based sampling methods. Trained researchers conducted interviews using computer-assisted techniques within the participants’ homes. For those who were unable or unwilling to participate personally, proxy respondents, generally a spouse or other family member, were interviewed. The data collected included sociodemographic characteristics, health status and diagnosis, and health behaviors.

Due to variations in data availability across countries and waves, we adopted a two-phase approach with 4-year windows: an initial phase covering 2011–2015 (start phase) and a subsequent phase covering 2015–2019 (end phase). The rationale for this two-phase approach was to capture significant policy and practice changes in dementia care across Europe during this period. The interval between these phases coincided with several important developments in dementia care: (1) the implementation of the World Health Organization’s Global Action Plan on the Public Health Response to Dementia 2017–2025, which established targets for diagnosis rates [6]; (2) the launch or updating of national dementia strategies in several European countries following the EU Joint Action on Dementia (2016) [21–24]; and (3) increased public awareness campaigns and clinical training initiatives aimed at improving early diagnosis. These policy and practice developments could potentially influence diagnosis rates through improved detection pathways, more standardized diagnostic criteria, enhanced professional education, and reduced stigma. Given these factors, we hypothesized that the underdiagnosis rate would reduce in most of the European countries. This strategy ensured capture of variables required for this analysis for all 19 countries, which allowed for direct cross-national comparisons.

While SHARE is designed as a longitudinal survey where participants may contribute to multiple waves, our analytical approach treats the data as repeated cross-sectional rather than focusing on within-individual changes. This approach is appropriate because: (1) SHARE incorporates refreshment samples in each wave to maintain representativeness, especially to account for population aging; (2) appropriate survey weights are applied to ensure each phase represents the target population; and (3) our research question focuses on population-level trends rather than individual-level changes in diagnosis status. This repeated cross-sectional approach has been successfully employed in previous SHARE-based research examining temporal trends across European countries [25].

This analysis only included participants aged 60 or above and had a cognition score above established cut-off for probable dementia (further details provided in the outcomes section) or had a diagnosed dementia. Finally, a total of 10,420 individuals were included in the study. The data are publicly available. The use of secondary de-identified data made this study exempt from institutional review board review. Participants in each wave gave informed consent and each survey was approved by both the Ethics Council of the Max Planck Society and ethics committees in participating countries [26].

### Outcome

#### Underdiagnosis of probable dementia

Individuals were considered having underdiagnosis of dementia if they had probable dementia but no confirmed diagnosis. This created a binary outcome variable (yes/no) for underdiagnosis based on the presence of probable dementia and absence of confirmed diagnosis. Probable dementia was judged based on cognition scores, derived from a validated algorithm designed for HRS-based studies of dementia [27, 28]. The algorithm incorporates performance scores of cognitive status for self-reported and for proxy-reported participants, respectively. Self-reported score in SHARE was evaluated by a 25-point cognitive scale that includes an immediate10-noun free recall test (ranged 0–10 points), delayed 10-noun free recall test (ranged 0–10 points), and a serial five subtraction test (ranged 0–5 points). The proxy-reported score is a 9-point scale, covering the memory function (ranged 0–4 points) and limitations in five instrumental activities of daily living (IADLs) (ranged 0–5 points). Previous studies have suggested that older individuals, males, and those responded by proxy typically exhibit lower cognitive performance [29, 30]. In this study, probable dementia was defined using stratified cognitive score thresholds. Participants were first categorized into subgroups based on age (60–64, 65–69, 70–74, 75–79, 80–84, 85–89, ≥ 90 years), sex (male, female), education level (primary or below, secondary, post-secondary or above), and proxy status (yes, no). Within each subgroup, individuals were classified as having probable dementia if their cognition score was below 1.5 standard deviations (SDs) from that subgroup’s mean score [28]. This approach accounted for demographic variations in cognitive performance. Confirmed diagnosis was obtained by the question “Has a doctor ever told you that you had or currently have Alzheimer’s disease, dementia, organic brain syndrome, senility or any other serious memory impairment?”, with response of yes or no.

### Potential predictors

In this study, we identified potential predictors based on the Andersen Healthcare Utilization Model alongside the Socio-Ecological Model. The Andersen model, used for forecasting and elucidating the use of health services, categorized predictors into three distinct domains: predisposing factors (e.g., age, sex, and education), enabling factors (e.g., health insurance and healthcare accessibility), and need factors [31]. Need factors were not considered in our study as they serve as proxies of dementia. Complementing this, the Socio-Ecological Model underscores the multifaceted influences on health behaviors across individual, interpersonal, organizational, community, and public policy dimensions [32]. This model assists the identification of factors in different aspects, including social support, healthcare system barriers, and policy elements that affect access to healthcare and dementia treatment. By synthesizing these frameworks, we classified the potential predictors into two main categories: individual-level and country-level predictors.

Individual-level factors included the sociodemographic factors (age, sex, marital status [married or cohabited, single or divorced or widowed], and education attainment [primary or lower, secondary, post-secondary or above]), household income, rurality of residence (rural, town, city or suburbs), living in nursing home (yes, no), retirement status (yes, no), number of children (none, one, at least two), hearing impairment (yes, no), self-reported chronic conditions except for dementia (none, one, at least two), utilization of outpatient care in the last 12 months (yes, no), utilization of inpatient care in the last 12 months (yes, no), and health insurance status (covered, not covered). Household income was self-reported gross total household income and was divided into five quintiles for analysis, with quintiles calculated within each country and across all survey participants (including those not screened positive for probable dementia).

Country-level factors included 11 measures of affordability and availability of resources for services, extracted from the Eurostat, the statistical office run by the European Commission and the official provider of statistics at the European level [33]. The measures were (1) general practitioners per 1000 inhabitants, (2) psychiatrists per 1000 inhabitants, (3) practicing nurses per 1000 inhabitants, (4) formal long-term care (LTC) workers at home per 100 inhabitants aged 65 and over, (5) formal LTC workers at institutions per 100 inhabitants aged 65 and over, (6) gross national income per capita (1000 US dollars), (7) current health expenditure per capita (1000 US dollars), (8) long-term care beds per 1000 inhabitants aged 65 and over, (9) psychiatric hospital beds per 1000 inhabitants aged 65 and over, (10) computed tomography (CT) scanners per million inhabitants, (11) magnetic resonance imaging (MRI) units, per million inhabitants, and (12) positron emission tomography (PET) scanners, per million inhabitants.

### Statistical analysis

The basic characteristics were described as number (percentage) for categorical variables and as mean (standard deviation, SD) for continuous variables. The differences were compared using chi-square test for categorical variables and ANOVA for continuous variables. The percentage of potential diagnosis was described at start and end phase, respectively, with 95% confidence interval (CIs) reported.

To explore the temporal trends and factors associated with underdiagnosis of dementia, we used weighted logistic regression models computed based on survey weighting, which was used to adjust for the complex survey design, including the unequal probability of selection, clustering, and stratification, to make estimates representative of each country. The weight values were provided in the SHARE datasets. Details of weight calculation are reported elsewhere [34].

**Fig. 1:**
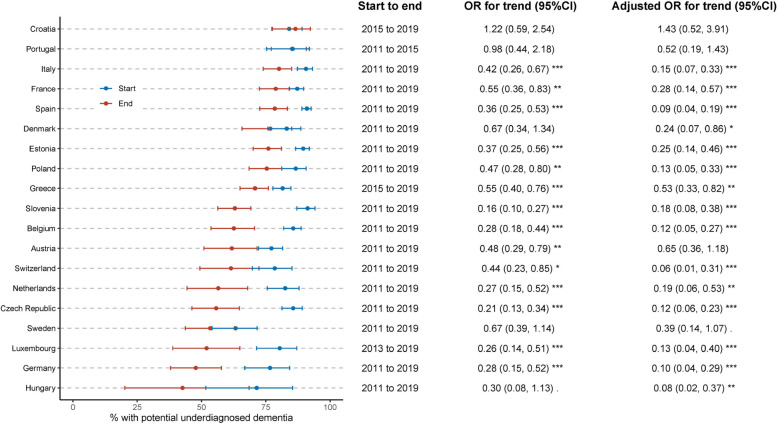
Percentage of having potential underdiagnosis of dementia by phase. Odds ratios (ORs) and their 95% confidence intervals (CIs) were estimated from weighted logistic regression, with underdiagnosis of dementia as the dependent variable and phase (end vs start [reference]) as the predictor. Adjusted ORs (with 95% CIs) were estimated from the model controlling for age, sex, education attainment, household income, marital status, retirement status, number of children, rurality of residence, living in nurse home, number of chronic conditions, hearing loss, and proxy status. OR > 1 indicates a higher likelihood of experiencing potential underdiagnosis of dementia during the end phase compared to the start phase. *** *P* < 0.001; ** *P* < 0.01; * *P* < 0.05;. *P* < 0.1

We conducted several weighted logistic regression models:

1. Country-specific models (Fig. 1): One model per country was constructed to estimate temporal trends of the rate of dementia underdiagnosis. The phase (start phase [reference] vs end phase) was the predictor, adjusting for individual-level factors (age, sex, education attainment, household income, marital status, retirement status, number of children, rurality of residence, living in nurse home, number of chronic conditions, hearing loss, utilization of outpatient care, utilization of inpatient care, health insurance status, and proxy status). Country-level factors were not included in these models as they were country-specific.
2. Combined individual-level and country-level model: This model included both individual-level and country-level factors simultaneously in a single regression, with underdiagnosis of probable dementia as the dependent variable. This approach allows us to assess the independent contributions of both individual- and country-level factors while accounting for their mutual influences. The model included all individual-level factors listed above, all country-level factors (general practitioners, psychiatrists, practicing nurses, formal LTC workers at home or institutions, gross national income, current health expenditure, long-term care beds, psychiatric hospital beds, CT scanners, MRI units, PET scanners), and the variable “country” to account for any residual country-specific effects not captured by the measured country-level factors.
3. Interaction model: To explore factors contributing to temporal trends of underdiagnosis, we added interaction terms between the phase variable and each predictor (one at a time) to the combined model above. This approach avoids potential collinearity issues that would arise from including all interactions simultaneously.

To explore potential variations in temporal trend by age, sex, and income level, we conducted two additional analyses:

1. Subgroup analyses: For each subgroup (defined by age groups, sex, and household income levels), we conducted separate weighted logistic regression models. These models included phase as the predictor while adjusting for all other individual-level factors. Country-level factors were not included in these subgroup analyses as they were conducted separately for each country. Due to small sample sizes in some subgroups, particularly in the 90 + age group and highest income quintile for several countries, some estimates could not be reliably calculated.
2. Interaction analyses: To formally test whether age, sex, or income modified the temporal trends, we included interaction terms between phase and each subgroup factor (e.g., phase × age group) in the country-specific models, adjusting for other individual-level factors.

It is important to note that for the analysis of chronic conditions, we observed no cases with zero chronic conditions and no potential underdiagnosis of dementia. To avoid statistical issues with perfect prediction and inflated coefficients, we randomly reclassified two cases originally belonging to the categories “1 chronic condition” or “2 + chronic conditions” (with no potential dementia underdiagnosis) into the “no chronic condition” category.

Adjusted odds ratios (aORs) and 95% CI were reported for each model. All analyses were conducted in R (version 4.3.0). Statistical significance was set at *P* < 0.05.

## Results

A total of 10,420 participants from 19 countries were included in this analysis over the two phases. The start phase (2011–2015) included 929 participants without underdiagnosis of potential dementia and 6077 with underdiagnosis of potential dementia. The end phase (2015–2019) included 986 participants without underdiagnosis of potential dementia and 2428 with underdiagnosis of potential dementia.

### Basic characteristics

Table 1 shows the basic characterizes of included participants. Among all participants underdiagnosed with probable dementia over the two phases, almost half (40.6%) were aged 70–79. The majority of this group were females (58.8%) and with primary education or below (59.6%). About one-third (29.1%) were in the lowest quintile of income while 10.8% were in the highest quintile. When comparing participants with and without underdiagnosis of probable dementia, there were significant differences in age (*P* < 0.001), education (*P* < 0.001), marital status (*P* = 0.005), rurality of residence (*P* = 0.026), residence in a nursing home (*P* < 0.001), number of chronic conditions (*P* < 0.001), hearing loss (*P* = 0.001), utilization of inpatient care (*P* < 0.001), and proxy status (*P* < 0.001). No significant differences were noted for sex, household income, number of children, utilization of outpatient care, and health insurance status.

**Table 1:** Basic characteristics of included participants. Categorical variables are reported as number (percentage), and continuous variables are reported as number (percentage). Wealth quintiles are calculated with respect to all survey participants (including those not screening positive for depressive symptoms). *P* values reflect unweighted group comparison and were extracted from two-tailed *t*-tests (for continuous variables) and chi-square tests (for categorical variables)

Variable	**Start phase (2011–2015) (*n* = 7005)**			**End phase (2015–2019) (*n* = 3397)**			**Overall (*n* = 10,402)**		
	Without potential underdiagnosis (*n* = 929)	With potential underdiagnosis (*n* = 6077)	*P*	Without potential underdiagnosis (*n* = 986)	With potential underdiagnosis (*n* = 2428)	*P*	Without potential underdiagnosis (*n* = 1915)	With potential underdiagnosis (*n* = 8505)	*P*
	*n (%)*	*n (%)*		*n (%)*	*n (%)*		*n (%)*	*n (%)*	
**Age, mean (SD)**	79.24 (8.21)	75.30 (7.77)	< 0.001	81.13 (8.25)	79.01 (8.39)	< 0.001	80.22 (8.28)	76.36 (8.13)	< 0.001
60–69	122 (13.1%)	1492 (24.6%)	< 0.001	113 (11.5%)	370 (15.2%)	< 0.001	235 (12.3%)	1862 (21.9%)	< 0.001
70–79	316 (34.0%)	2661 (43.8%)		256 (26.0%)	789 (32.5%)		572 (29.9%)	3450 (40.6%)	
80–89	409 (44.0%)	1771 (29.1%)		454 (46.0%)	1039 (42.8%)		863 (45.1%)	2810 (33.0%)	
≥ 90	82 (8.8%)	153 (2.5%)		163 (16.5%)	230 (9.5%)		245 (12.8%)	383 (4.5%)	
**Sex (= female)**	576 (62.0%)	3441 (56.6%)	0.002	579 (58.7%)	1563 (64.4%)	0.002	1155 (60.3%)	5004 (58.8%)	0.245
**Education attained**									
Primary or lower	478 (51.5%)	3575 (58.8%)	< 0.001	398 (40.4%)	1498 (61.7%)	< 0.001	876 (45.7%)	5073 (59.6%)	< 0.001
Secondary	346 (37.2%)	2067 (34.0%)		412 (41.8%)	778 (32.0%)		758 (39.6%)	2845 (33.5%)	
Post-secondary or above	105 (11.3%)	435 (7.2%)		176 (17.8%)	152 (6.3%)		281 (14.7%)	587 (6.9%)	
**Household income**									
Lowest (Q1)	283 (30.5%)	1627 (26.8%)	0.129	295 (29.9%)	846 (34.8%)	< 0.001	578 (30.2%)	2473 (29.1%)	0.422
Q2	217 (23.4%)	1404 (23.1%)		224 (22.7%)	677 (27.9%)		441 (23.0%)	2081 (24.5%)	
Q3	180 (19.4%)	1247 (20.5%)		197 (20.0%)	436 (18.0%)		377 (19.7%)	1683 (19.8%)	
Q4	139 (15.0%)	1044 (17.2%)		153 (15.5%)	304 (12.5%)		292 (15.2%)	1348 (15.8%)	
Highest (Q5)	110 (11.8%)	755 (12.4%)		117 (11.9%)	165 (6.8%)		227 (11.9%)	920 (10.8%)	
**Marital status**									
Married, cohabitating, or living with partner	462 (49.7%)	3535 (58.2%)	< 0.001	551 (55.9%)	1270 (52.3%)	0.063	1013 (52.9%)	4805 (56.5%)	0.005
Single, divorced, or widowed	467 (50.3%)	2542 (41.8%)		435 (44.1%)	1158 (47.7%)		902 (47.1%)	3700 (43.5%)	
**Retired (= yes)**	712 (76.6%)	4694 (77.2%)	0.684	827 (83.9%)	1890 (77.8%)	< 0.001	1539 (80.4%)	6584 (77.4%)	0.005
**Number of children**									
None	94 (10.1%)	551 (9.1%)	0.192	68 (6.9%)	241 (9.9%)	0.015	162 (8.5%)	792 (9.3%)	0.475
One	194 (20.9%)	1158 (19.1%)		179 (18.2%)	454 (18.7%)		373 (19.5%)	1612 (19.0%)	
At least two	641 (69.0%)	4368 (71.9%)		739 (74.9%)	1733 (71.4%)		1380 (72.1%)	6101 (71.7%)	
**Rurality of residence**									
City or suburbs of city	249 (26.8%)	1336 (22.0%)	0.004	219 (22.2%)	511 (21.0%)	0.373	468 (24.4%)	1847 (21.7%)	0.026
Town	372 (40.0%)	2528 (41.6%)		400 (40.6%)	951 (39.2%)		772 (40.3%)	3479 (40.9%)	
Rural or village	308 (33.2%)	2213 (36.4%)		367 (37.2%)	966 (39.8%)		675 (35.2%)	3179 (37.4%)	
**Living in nursing home (= yes)**	68 (7.3%)	124 (2.0%)	< 0.001	106 (10.8%)	122 (5.0%)	< 0.001	174 (9.1%)	246 (2.9%)	< 0.001
**Number of chronic conditions**									
None	0 (0%)	703 (11.6%)	< 0.001	0 (0%)	209 (8.6%)	< 0.001	0 (0%)	912 (10.7%)	< 0.001
One	140 (15.1%)	1559 (25.7%)		121 (12.3%)	538 (22.2%)		261 (13.6%)	2097 (24.7%)	
At least two	789 (84.9%)	3815 (62.8%)		865 (87.7%)	1681 (69.2%)		1654 (86.4%)	5496 (64.6%)	
**Hearing loss (= yes)**	120 (12.9%)	578 (9.5%)	0.002	130 (13.2%)	298 (12.3%)	0.502	250 (13.1%)	876 (10.3%)	0.001
**Past-year outpatient visit (= yes)**	517 (55.7%)	4167 (68.6%)	< 0.001	933 (94.6%)	2207 (90.9%)	< 0.001	1450 (75.7%)	6374 (74.9%)	0.498
**Past-year inpatient visit (= yes)**	275 (29.6%)	1107 (18.2%)	< 0.001	271 (27.5%)	539 (22.2%)	0.001	546 (28.5%)	1646 (19.4%)	< 0.001
**Covered by health insurance (= yes)**	893 (96.1%)	5876 (96.7%)	0.427	986 (100.0%)	2426 (99.9%)	0.904	1879 (98.1%)	8302 (97.6%)	0.210
**Proxy (= yes)**	561 (60.4%)	2544 (41.9%)	< 0.001	413 (42.0%)	164 (6.8%)	< 0.001	974 (50.9%)	2708 (31.9%)	< 0.001

### Trends of underdiagnosis across countries

The percentage of having underdiagnosis of probable dementia varied widely across countries (Fig. 1), ranging from 42.61% (95% CI 20.21, 68.51) in Hungary to 86.55% (95% CI 77.61–92.28) at the end phase (2015–2019). From the start to end phase, the underdiagnosis of probably dementia had a significant decline in 15 of 19 countries. We observed the smallest decrease in Greece (aOR = 0.53, 95% CI [0.33, 0.82]) and the largest decrease in Switzerland (aOR = 0.06, 95% CI [0.01, 0.31]) (Fig. 1). There was no significant change in Croatia, Portugal, Austria, and Sweden.

### Individual-level contributing factors

We identified individual-level factors associated with the risk of underdiagnosis of probable dementia (Table 1). As we hypothesized, the risk of underdiagnosis was lower in the end phase (2015–2019) than the start phase (2011–2015) (aOR = 0.22, 95% CI [0.16, 0.29]) (Table 2, column 2). Compared to those aged 70–79, those aged 80–89 (aOR = 0.65 [0.51, 0.82]) and ≥ 90 (aOR = 0.42 [0.30, 0.60]) were less likely to be underdiagnosed. Individuals with higher education (aOR = 0.46 [0.36, 0.58] secondary; aOR = 0.31 [0.20, 0.49] post-secondary vs primary or lower), residing in a nursing home (aOR = 0.42 [0.27, 0.66]), with multimorbidity (aOR = 0.45, 95% CI [0.35, 0.58] vs one chronic condition), and reported by a proxy (aOR = 0.14 [0.11, 0.18]) had lower risk of underdiagnosis of probable dementia (Table 2, column 2). Those who were married or cohabiting (aOR = 1.50, 95% CI [1.23, 2.06]), utilized outpatient care (aOR = 1.67, 95% CI [1.29, 2.16]), and without chronic condition (aOR = 94.29, 95% CI [27.98, 317.78]) were more likely to be undiagnosed (Table 2, column 2). Household income, retirement status, number of children, rurality of residence, hearing loss, inpatient care use, and health insurance coverage were not significant in analysis (Table 2, column 2).

**Table 2:** Individual-level predictors of potential underdiagnosis of dementia. Odds ratios (ORs) and their 95% confidence intervals (CIs) were estimated from weighted logistic regression, with underdiagnosis of dementia as the dependent variable and listed variables, country, and country-level factors listed in Table 3 as potential predictors. ORs for trend and their 95% CIs were estimated from the same models but added the interactions between listed variables and phase. “–” means not applicable. *** *P* < 0.001; ** *P* < 0.01; * *P* < 0.05;. *P* < 0.1

Individual-level variables	Adjusted OR (95% CI)	Interaction (adjusted OR for trend, 95% CI)
**Phase (= end)**	0.22 (0.16, 0.29)***	–
**Age**		
60–69	1.21 (0.84, 1.76)	0.94 (0.43, 2.05)
70–79	Reference	Reference
80–89	0.65 (0.51, 0.82)***	1.65 (1.02, 2.67)*
≥ 90	0.42 (0.30, 0.60)***	1.45 (0.69, 3.02)
**Sex (= male)**	1.14 (0.89, 1.45)	0.68 (0.42, 1.11)
**Education attained**		
Primary or lower	Reference	Reference
Secondary	0.46 (0.36, 0.58)***	0.59 (0.36, 0.96)*
Post-secondary or above	0.31 (0.20, 0.49)***	0.17 (0.07, 0.42)***
**Household income**		
Lowest (Q1)	Reference	Reference
Q2	1.17 (0.88, 1.54)	1.51 (0.86, 2.63)
Q3	0.92 (0.66, 1.27)	0.75 (0.38, 1.46)
Q4	1.25 (0.90, 1.74)	0.96 (0.48, 1.92)
Highest (Q5)	0.92 (0.60, 1.41)	1.91 (0.83, 4.40)
**Marital status (= married, cohabitating, or living with partner)**	1.59 (1.23, 2.06)***	0.83 (0.49, 1.41)
**Retired (= yes)**	0.92 (0.72, 1.19)	0.57 (0.33, 0.97)*
**Number of children**		
None	1.20 (0.83, 1.73)	1.51 (0.70, 3.23)
One	1.24 (0.93, 1.66)	1.32 (0.74, 2.36)
At least two	Reference	Reference
**Rurality of residence**		
City or suburbs of city	Reference	Reference
Town	1.22 (0.92, 1.62)	0.78 (0.44, 1.37)
Rural or village	1.21 (0.90, 1.61)	0.74 (0.41, 1.33)
**Living in nursing home (= yes)**	0.42 (0.27, 0.66)***	2.76 (1.30, 5.85)**
**Number of chronic conditions**		
None†	94.29 (27.98, 317.78)***	2.72 (1.37, 5.40)***
One	Reference	Reference
At least two	0.45 (0.35, 0.58)***	0.97 (0.57, 1.68)
**Hearing loss (= yes)**	1.18 (0.85, 1.62)	0.74 (0.39, 1.42)
**Past-year outpatient visit (= yes)**	1.67 (1.29, 2.16)***	0.49 (0.25, 0.94)*
**Past-year inpatient visit (= yes)**	0.88 (0.71, 1.09)	1.98 (1.28, 3.07)**
**Covered by health insurance (= yes)**	1.74 (0.72, 4.18)	–
**Proxy (= yes)**	0.14 (0.11, 0.18)***	0.15 (0.09, 0.26)***

^†^Given the absence of cases with zero chronic conditions and no potential underdiagnosis of dementia, as illustrated in Table 1, and to avoid an inflated coefficient, we randomly reclassified two cases originally belonging to the categories “1 chronic condition” or “2 chronic conditions” (with no potential dementia underdiagnosis) into the “no chronic condition” category

The interaction analysis identified factors that moderate the association between study phase (2011–2015 vs 2015–2019) and risk of underdiagnosis of probable (Table 2). These include age, education level, retirement status, nursing home residence, number of chronic diseases, outpatient and inpatient care use, and proxy status (Table 2, column 3). Over the two phases, there was greater reduction in underdiagnosis risk among those with higher education (aOR = 0.59 [0.36, 0.96] secondary; aOR = 0.17 [0.07, 0.42] post-secondary vs primary or lower), retired participants (aOR = 0.57 [0.33, 0.97]), those who utilized outpatient care (aOR = 0.49 [0.25, 0.94]), and proxy-reported respondents (aOR = 0.15 [0.09, 0.26]) (Table 2, column 3). Conversely, there was less risk reduction in individuals who are older (aOR = 1.65 [1.02, 2.67] for 80–89 vs 70–79 years), living in a nursing home (aOR = 2.76 [1.30, 5.85]), without chronic condition (aOR = 2.72 [1.37, 5.40]), and had utilized inpatient care (aOR = 1.98 [1.28, 3.07]) (Table 2, column 3).

### Country-level contributing factors

At a country-level column 2 (Table 3), having more general practitioners per 1000 inhabitants (aOR = 1.35 [1.03, 1.76]), more formal LTC workers at home (aOR = 1.09 [1.03, 1.16]), and more PET scanners (aOR = 1.16 [1.03, 1.30]) was associated with increased risks of underdiagnosis of probable dementia. Conversely, having more psychiatrists (aOR = 0.09 [0.02, 0.46]), more practicing nurses (aOR = 0.91 [0.88, 0.95]), and more psychiatric hospital beds (aOR = 0.51 [0.38, 0.68]) (Table 3, column 2) predicted reduced risks. Furthermore, there was a greater temporal decline (from start to end phase) in countries with more psychiatrists (aOR = 0.01 [0.00, 0.28]), more formal LTC worker at home (aOR = 0.86 [0.77, 0.96]) or at institutions (aOR = 0.88 [0.79, 0.97]), and more PET scanners (aOR = 0.71 [0.55, 0.92]) (Table 3, column 3).

**Table 3:** Country-level predictors of potential underdiagnosis of dementia. Odds ratios (ORs) and their 95% confidence intervals (CIs) were estimated from weighted logistic regression, with underdiagnosis of dementia as the dependent variable, listed variables, country, and the individual-levels factors listed in Table 2 as potential predictors. ORs for trend and their 95% CIs were estimated from the same models but added the interactions between listed variables and phase. *** *P* < 0.001; ** *P* < 0.01; * *P* < 0.05;. *P* < 0.1

National-level variables	Adjusted OR (95% CI)	Interaction (adjusted OR for trend, 95% CI)
General practitioners per 1000 inhabitants	1.35 (1.03, 1.76)*	1.41 (0.82, 2.43)
Psychiatrists per 1000 inhabitants	0.09 (0.02, 0.46)**	0.01 (0.00, 0.28)**
Practicing nursing per 1000 inhabitants	0.91 (0.88, 0.95)***	0.92 (0.85, 1.00)
Formal LTC workers at home, per 100 inhabitants aged 65 and over	1.09 (1.03, 1.16)**	0.86 (0.77, 0.96)**
Formal LTC workers at institutions, per 100 inhabitants aged 65 and over	1.00 (0.95, 1.06)	0.88 (0.79, 0.97)*
Gross national income per capita (1000 US dollars)†	1.00 (1.00, 1.00)	1.00 (1.00, 1.00)***
Current health expenditure per capita (1000 US dollars)†	1.00 (1.00, 1.00)	1.00 (1.00, 1.00)**
Long-term care beds, per 1000 inhabitants aged 65 and over	0.99 (0.96, 1.01)	1.04 (0.97, 1.12)
Psychiatric hospital beds per 1000 inhabitants aged 65 and over	0.51 (0.38, 0.68)***	0.64 (0.36, 1.14)
Computed tomography scanners, per million inhabitants	1.00 (0.99, 1.01)	0.99 (0.97, 1.01)
Magnetic resonance imaging units, per million inhabitants	1.00 (0.98, 1.01)	0.98 (0.95, 1.01)
Positron emission tomography scanners, per million inhabitants	1.16 (1.03, 1.30)*	0.71 (0.55, 0.92)**

^†^The coefficients for gross national income per capita and current health expenditure per capita are presented as 1.00 (1.00, 1.00) due to rounding but represent statistically significant effects

### Subgroup analyses

In subgroup analysis of temporal trends by age (Table 4 column 5), the underdiagnosis rate had decreased significantly in most age groups (60–69, 70–79, 80–89 years) in seven countries. For the 60–69 age group, we observed the smallest decrease in Slovenia (aOR = 0.13 [0.03, 0.49]) and the greatest decrease in Germany (aOR = 0.03 [0.00, 0.22]). For the 70–79 group, Poland showed the smallest decrease (aOR = 0.34 [0.13, 0.92]) while Slovenia and Germany demonstrated the largest (aOR = 0.12 [0.05, 0.29] and aOR = 0.12 [0.03, 0.40], respectively). For those aged 80–89, France had the smallest decrease (AOR = 0.53 [0.28, 0.97]) and Czech Republic had the largest (aOR = 0.19 [0.08, 0.44]). No countries showed significant trend decreases in the age group of 90 or above. However, in Sweden, this group experienced an increasing trend over time (aOR = 10.89 [1.89, 62.85]). After including an interaction term between phase and age (Table 4, column 6), we found that those aged 89–90 experienced a slower decline in Germany (aOR = 4.70 [1.03, 21.37]), compared to those aged 70–79. A similar trend was observed in those aged at least 90 in France (aOR = 5.81 [1.36, 24.76]) and Sweden (aOR = 6.96 [1.01, 47.81]).

**Table 4:** Percentage having potential underdiagnosis of dementia by phase and age. Odds ratios (ORs) and their 95% confidence intervals (CIs) I were estimated from weighted logistic regression models for each age group, with underdiagnosis of dementia as the dependent variable and phase (end, vs start [reference]) as the predictor, controlling for sex, education attainment, household income, marital status, retirement status, number of children, rurality of residence, living in nurse home, number of chronic conditions, hearing loss, utilization of outpatient care, utilization of inpatient care, health insurance status, and proxy status. ORs and their 95% CIs II were estimated from weighted logistic regression models for all age groups, with underdiagnosis of dementia as the dependent variable and interaction between phase (end, vs start [reference]) and age as the predictor, controlling for sex, education attainment, household income, marital status, retirement status, number of children, rurality of residence, living in nurse home, number of chronic conditions, hearing loss, utilization of outpatient care, utilization of inpatient care, health insurance status, and proxy status. “–” means not applicable or unable to estimated due to lacking of corresponding samples. *** *P* < 0.001; ** *P* < 0.01; * *P* < 0.05;. *P* < 0.1

Country	Age	Start phase	End phase	Interaction I Adjusted OR for trend (95% CI)	Interaction II Adjusted OR for trend (95% CI)
Austria	60–69	80.3 (70.6, 87.4)	82.1 (38.7, 97.1)	0.75 (0.17, 3.21)	2.53 (0.35, 18.42)
	70–79	84.2 (77.4, 89.3)	71.4 (48.6, 86.9)	0.39 (0.14, 1.11)	Reference
	80–89	69.1 (58.9, 77.8)	64.9 (48.9, 78.2)	0.80 (0.36, 1.78)	1.89 (0.52, 6.79)
	≥ 90	71.2 (38.2, 90.8)	37.0 (17.6, 61.8)	0.27 (0.04, 1.85)	0.50 (0.08, 3.29)
Belgium	60–69	95.1 (87.2, 98.2)	70.9 (36.3, 91.2)	0.12 (0.02, 0.74)*	0.53 (0.08, 3.31)
	70–79	87.6 (81.8, 91.8)	64.3 (46.3, 79.0)	0.28 (0.12, 0.67)**	Reference
	80–89	82.7 (76.0, 87.9)	65.5 (53.4, 75.8)	0.37 (0.19, 0.73)**	1.67 (0.56, 4.94)
	≥ 90	63.0 (36.6, 83.4)	44.8 (23.6, 68.2)	1.08 (0.22, 5.34)	2.36 (0.48, 11.66)
Croatia	60–69	84.7 (70.7, 92.6)	91.7 (67.7, 98.3)	2.96 (0.36, 24.25)	2.71 (0.35, 20.96)
	70–79	84.8 (74.4, 91.5)	80.5 (62.6, 91.0)	0.83 (0.26, 2.63)	Reference
	80–89	83.1 (70.1, 91.2)	89.2 (72.4, 96.3)	1.36 (0.32, 5.85)	2.22 (0.37, 13.29)
	≥ 90	–	–	–	–
Czech Republic	60–69	85.3 (74.5, 92.0)	76.5 (52.6, 90.5)	0.52 (0.14, 1.93)	2.47 (0.56, 10.81)
	70–79	87.2 (80.8, 91.7)	59.5 (43.5, 73.7)	0.23 (0.10, 0.50)***	Reference
	80–89	83.4 (74.4, 89.7)	52.6 (39.3, 65.5)	0.19 (0.08, 0.44)***	0.97 (0.33, 2.86)
	≥ 90	–	–	–	–
Denmark	60–69	100.0 (100.0, 100.0)	–	–	–
	70–79	79.3 (66.6, 88.0)	77.9 (56.4, 90.5)	0.73 (0.22, 2.51)	Reference
	80–89	79.3 (65.1, 88.7)	78.4 (61.8, 89.0)	1.01 (0.33, 3.07)	1.10 (0.23, 5.32)
	≥ 90	91.8 (51.5, 99.2)	59.0 (23.1, 87.3)	0.15 (0.01, 3.47)	0.14 (0.01, 2.21)
Estonia	60–69	93.9 (86.4, 97.4)	93.6 (74.5, 98.6)	1.23 (0.19, 7.96)	4.36 (0.68, 27.88)
	70–79	91.1 (87.0, 93.9)	69.5 (57.6, 79.2)	0.22 (0.11, 0.43)***	Reference
	80–89	86.6 (80.5, 91.0)	76.3 (67.3, 83.4)	0.44 (0.22, 0.88)*	2.14 (0.85, 5.42)
	≥ 90	77.7 (45.1, 93.6)	63.6 (43.5, 79.9)	0.21 (0.02, 2.88)	2.21 (0.46, 10.64)
France	60–69	94.3 (88.3, 97.3)	93.3 (75.2, 98.5)	0.73 (0.16, 3.25)	1.57 (0.24, 10.41)
	70–79	89.6 (84.8, 93.0)	82.2 (66.3, 91.6)	0.50 (0.18, 1.36)	Reference
	80–89	85.7 (80.3, 89.8)	76.1 (66.4, 83.7)	0.53 (0.28, 0.97)*	0.99 (0.32, 3.06)
	≥ 90	45.1 (24.6, 67.4)	72.7 (55.4, 85.1)	3.11 (0.96, 10.10)	5.81 (1.36, 24.76)*
Germany	60–69	93.8 (76.7, 98.6)	42.4 (16.7, 72.9)	0.03 (0.00, 0.22)***	0.41 (0.04, 3.74)
	70–79	78.2 (62.1, 88.7)	33.0 (16.1, 55.9)	0.12 (0.03, 0.40)***	Reference
	80–89	68.6 (49.4, 83.1)	57.2 (43.8, 69.7)	0.59 (0.21, 1.65)	4.70 (1.03, 21.37)*
	≥ 90	–	32.6 (11.6, 64.1)	–	–
Greece	60–69	95.5 (89.5, 98.2)	85.2 (64.4, 94.8)	0.31 (0.08, 1.24)	0.44 (0.09, 2.13)
	70–79	82.3 (75.8, 87.3)	72.9 (61.5, 81.9)	0.59 (0.32, 1.09)	Reference
	80–89	81.2 (75.2, 86.0)	71.1 (63.2, 78.0)	0.56 (0.35, 0.90)*	0.97 (0.44, 2.12)
	≥ 90	44.1 (25.7, 64.3)	53.2 (32.5, 72.8)	1.14 (0.34, 3.87)	2.41 (0.69, 8.41)
Hungary	60–69	43.9 (14.9, 77.7)	13.9 (1.9, 57.1)	0.44 (0.02, 7.96)	1.62 (0.10, 27.40)
	70–79	81.9 (65.0, 91.7)	56.7 (21.1, 86.5)	0.21 (0.03, 1.25)	Reference
	80–89	81.4 (59.8, 92.8)	58.9 (23.6, 86.9)	0.34 (0.05, 2.15)	1.20 (0.10, 13.92)
	≥ 90	–	–	–	–
Italy	60–69	97.0 (92.2, 98.9)	93.6 (76.2, 98.5)	0.27 (0.02, 3.30)	1.06 (0.15, 7.75)
	70–79	94.0 (89.9, 96.5)	87.2 (77.8, 93.0)	0.49 (0.20, 1.19)	Reference
	80–89	87.4 (80.3, 92.3)	78.0 (68.4, 85.3)	0.49 (0.24, 0.99)*	1.15 (0.36, 3.65)
	≥ 90	67.0 (39.0, 86.6)	51.3 (28.2, 73.9)	0.50 (0.10, 2.38)	1.17 (0.23, 5.96)
Luxembourg	60–69	94.0 (82.1, 98.2)	58.1 (33.1, 79.6)	0.07 (0.02, 0.34)**	0.22 (0.03, 1.51)
	70–79	80.8 (64.6, 90.7)	57.2 (35.1, 76.7)	0.30 (0.09, 1.04)	Reference
	80–89	72.7 (53.8, 85.9)	58.0 (33.1, 79.5)	0.52 (0.14, 1.95)	1.40 (0.23, 8.45)
	≥ 90	–	–	–	–
Netherlands	60–69	94.9 (80.5, 98.8)	50.3 (19.4, 80.9)	0.05 (0.01, 0.42)**	0.18 (0.02, 1.59)
	70–79	86.9 (76.8, 93.0)	63.3 (45.1, 78.4)	0.23 (0.08, 0.67)**	Reference
	80–89	73.0 (57.6, 84.3)	56.8 (36.5, 75.1)	0.64 (0.22, 1.87)	1.89 (0.44, 8.24)
	≥ 90	71.8 (34.0, 92.6)	–	–	–
Poland	60–69	93.4 (80.3, 98.0)	88.0 (73.1, 95.2)	0.54 (0.10, 2.78)	1.13 (0.18, 7.25)
	70–79	89.1 (79.9, 94.4)	78.5 (66.4, 87.1)	0.34 (0.13, 0.92)*	Reference
	80–89	77.3 (65.7, 85.9)	72.4 (59.9, 82.2)	0.70 (0.30, 1.62)	1.71 (0.51, 5.74)
	≥ 90	–	59.3 (37.4, 78.1)	–	–
Portugal	60–69	86.8 (65.5, 95.8)	88.1 (72.9, 95.4)	0.89 (0.29, 2.73)	2.14 (0.29, 15.88)
	70–79	95.0 (85.8, 98.3)	93.3 (86.7, 96.8)	0.67 (0.16, 2.86)	Reference
	80–89	70.8 (47.6, 86.6)	78.0 (60.7, 89.1)	0.66 (0.16, 2.69)	1.84 (0.28, 12.30)
	≥ 90	–	80.6 (34.7, 97.0)	–	–
Slovenia	60–69	89.2 (72.9, 96.2)	53.5 (35.5, 70.7)	0.13 (0.03, 0.49)**	1.15 (0.24, 5.63)
	70–79	95.7 (91.6, 97.9)	70.1 (56.7, 80.7)	0.12 (0.05, 0.29)***	Reference
	80–89	84.5 (74.2, 91.2)	63.4 (53.7, 72.0)	0.34 (0.16, 0.71)**	2.81 (0.86, 9.21)
	≥ 90	–	58.5 (36.8, 77.4)	–	–
Spain	60–69	97.5 (94.7, 98.8)	84.8 (64.8, 94.4)	0.12 (0.03, 0.48)**	0.31 (0.06, 1.57)
	70–79	94.3 (91.7, 96.2)	89.2 (78.4, 95.0)	0.44 (0.17, 1.12)	Reference
	80–89	82.3 (77.5, 86.3)	75.5 (65.4, 83.4)	0.62 (0.35, 1.12)	1.39 (0.46, 4.18)
	≥ 90	71.9 (48.7, 87.4)	63.6 (48.4, 76.5)	0.53 (0.15, 1.88)	1.50 (0.35, 6.46)
Sweden	60–69	82.8 (50.7, 95.8)	37.7 (9.5, 77.7)	0.16 (0.01, 1.77)	0.26 (0.03, 2.40)
	70–79	75.4 (57.6, 87.3)	69.0 (50.2, 83.1)	0.75 (0.25, 2.28)	Reference
	80–89	67.3 (54.1, 78.2)	49.0 (36.0, 62.1)	0.46 (0.21, 1.01)	0.68 (0.18, 2.66)
	≥ 90	22.0 (6.4, 53.8)	53.0 (28.5, 76.2)	10.89 (1.89, 62.85)**	6.96 (1.01, 47.81)*
Switzerland	60–69	92.5 (72.4, 98.3)	54.4 (23.0, 82.6)	0.12 (0.02, 0.75)*	0.44 (0.04, 4.68)
	70–79	81.4 (63.0, 91.8)	47.7 (20.3, 76.7)	0.26 (0.05, 1.29)	Reference
	80–89	71.0 (56.7, 82.0)	70.8 (51.4, 84.7)	1.22 (0.44, 3.44)	4.82 (0.79, 29.26)
	≥ 90	83.3 (46.6, 96.6)	59.4 (31.7, 82.2)	0.27 (0.04, 1.70)	1.46 (0.14, 15.73)

By sex (Table 5 column 5), the underdiagnosis rate had significantly declined among females in 13 of 19 countries and among males in nine countries. Among females, Greece had the smallest decrease (aOR = 0.52 [0.30, 0.91]) while Switzerland experienced the greatest decrease (aOR = 0.00 [0.00, 0.14]). Among males, we observed the smallest decrease in France (aOR = 0.23 [0.07, 0.73]) and the greatest decrease in Luxembourg (aOR = 0.00 [0.00, 0.03]). Compared to females (Table 5, column 6), males had more reduction in risks in Austria (aOR = 0.33 [0.11, 0.94]), Belgium (aOR = 0.11 [0.03, 0.33]), Luxembourg (aOR = 0.09 [0.01, 0.55]), and the Netherlands (aOR = 0.09 [0.02, 0.39]).

**Table 5:** Percentage having potential underdiagnosis of dementia by phase and sex. Odds ratios (ORs) and their 95% confidence intervals (CIs) I were estimated from weighted logistic regression models for each sex, with underdiagnosis of dementia as the dependent variable and phase (end, vs start [reference]) as the predictor, controlling for age, education attainment, household income, marital status, retirement status, number of children, rurality of residence, living in nurse home, number of chronic conditions, hearing loss, utilization of outpatient care, utilization of inpatient care, health insurance status, and proxy status. ORs and their 95% CIs II were estimated from weighted logistic regression models for all sexes, with underdiagnosis of dementia as the dependent variable and interaction between phase (end, vs start [reference]) and sex as the predictor, controlling for age, education attainment, household income, marital status, retirement status, number of children, rurality of residence, living in nurse home, number of chronic conditions, hearing loss, utilization of outpatient care, utilization of inpatient care, health insurance status, and proxy status. *** *P* < 0.001; ** *P* < 0.01; * *P* < 0.05;. *P* < 0.1

Country	Gender	Start phase	End phase	Interaction I Adjusted OR for trend (95% CI)	Interaction II Adjusted OR for trend (95% CI)
Austria	Female	72.3 (64.8, 78.7)	66.0 (52.0, 77.8)	0.66 (0.28, 1.57)	Reference
	Male	83.0 (76.3, 88.1)	55.6 (38.0, 71.9)	0.57 (0.20, 1.66)	0.33 (0.11, 0.94)*
Belgium	Female	81.6 (76.0, 86.1)	71.1 (60.9, 79.5)	0.17 (0.06, 0.47)***	Reference
	Male	91.3 (86.5, 94.5)	41.3 (26.6, 57.6)	0.03 (0.01, 0.18)***	0.11 (0.03, 0.33)***
Croatia	Female	85.5 (77.2, 91.1)	85.5 (74.1, 92.4)	0.98 (0.40, 2.43)	Reference
	Male	79.5 (65.7, 88.7)	89.5 (70.8, 96.8)	2.10 (0.70, 6.31)	2.17 (0.50, 9.36)
Czech Republic	Female	85.2 (78.8, 89.9)	57.8 (45.4, 69.3)	0.12 (0.05, 0.29)***	Reference
	Male	86.4 (80.2, 90.9)	52.9 (38.0, 67.2)	0.05 (0.02, 0.18)***	0.59 (0.20, 1.72)
Denmark	Female	82.3 (71.6, 89.6)	74.8 (61.0, 84.9)	0.40 (0.06, 2.55)	Reference
	Male	84.1 (72.6, 91.4)	81.5 (59.2, 93.1)	0.00 (0.00, 1.27)	0.85 (0.16, 4.59)
Estonia	Female	87.9 (83.7, 91.2)	70.0 (62.0, 76.9)	0.15 (0.07, 0.34)***	Reference
	Male	92.3 (88.3, 95.1)	86.3 (78.1, 91.7)	0.59 (0.23, 1.52)	1.83 (0.70, 4.78)
France	Female	84.7 (80.2, 88.3)	79.3 (71.4, 85.5)	0.27 (0.11, 0.64)**	Reference
	Male	90.6 (86.4, 93.5)	77.9 (65.8, 86.5)	0.23 (0.07, 0.73)*	0.74 (0.26, 2.08)
Germany	Female	68.8 (53.4, 80.9)	52.7 (38.5, 66.5)	0.08 (0.01, 0.98)*	Reference
	Male	86.1 (75.5, 92.6)	40.7 (28.1, 54.7)	0.04 (0.01, 0.25)***	0.23 (0.04, 1.19)
Greece	Female	80.6 (75.9, 84.6)	72.5 (65.5, 78.7)	0.52 (0.30, 0.91)*	Reference
	Male	83.5 (77.0, 88.4)	66.6 (55.9, 75.9)	0.47 (0.19, 1.14)	0.89 (0.34, 2.32)
Hungary	Female	72.0 (44.3, 89.3)	60.7 (31.3, 84.0)	0.13 (0.02, 0.82)*	Reference
	Male	70.1 (46.5, 86.4)	28.8 (6.6, 69.7)	0.05 (0.00, 10.28)	0.90 (0.09, 8.92)
Italy	Female	90.7 (86.1, 93.9)	80.5 (72.6, 86.6)	0.09 (0.03, 0.29)***	Reference
	Male	90.5 (85.1, 94.0)	79.1 (69.1, 86.5)	0.32 (0.08, 1.22)	1.49 (0.43, 5.24)
Luxembourg	Female	74.3 (60.5, 84.6)	51.8 (33.0, 70.0)	0.12 (0.02, 0.85)*	Reference
	Male	87.3 (74.5, 94.1)	52.3 (34.5, 69.5)	0.00 (0.00, 0.03)**	0.09 (0.01, 0.55)**
Netherlands	Female	76.5 (64.7, 85.3)	60.0 (43.3, 74.6)	0.36 (0.08, 1.66)	Reference
	Male	88.4 (80.0, 93.6)	51.9 (33.7, 69.6)	0.01 (0.00, 0.13)**	0.09 (0.02, 0.39)**
Poland	Female	85.3 (77.4, 90.8)	73.3 (64.4, 80.6)	0.09 (0.02, 0.34)***	Reference
	Male	88.9 (81.4, 93.6)	79.9 (68.3, 88.0)	0.16 (0.03, 0.87)*	0.55 (0.14, 2.22)
Portugal	Female	85.1 (73.5, 92.2)	83.4 (72.8, 90.4)	0.44 (0.13, 1.46)	Reference
	Male	86.7 (56.9, 97.0)	90.6 (79.2, 96.0)	0.48 (0.05, 4.51)	1.16 (0.21, 6.49)
Slovenia	Female	91.5 (86.0, 95.0)	63.4 (54.6, 71.3)	0.15 (0.05, 0.43)***	Reference
	Male	90.6 (83.2, 94.9)	62.4 (51.7, 71.9)	0.15 (0.04, 0.57)**	0.89 (0.26, 3.11)
Spain	Female	90.8 (88.2, 92.9)	79.2 (71.8, 85.0)	0.08 (0.03, 0.22)***	Reference
	Male	91.3 (88.1, 93.7)	77.2 (66.4, 85.2)	0.07 (0.02, 0.29)***	0.97 (0.39, 2.40)
Sweden	Female	58.3 (44.9, 70.6)	49.9 (36.5, 63.4)	0.20 (0.04, 1.15)	Reference
	Male	69.2 (56.8, 79.3)	57.2 (43.5, 69.9)	0.46 (0.09, 2.38)	0.66 (0.17, 2.52)
Switzerland	Female	75.4 (62.7, 84.9)	64.0 (48.6, 76.9)	0.00 (0.00, 0.14)**	Reference
	Male	82.5 (69.7, 90.6)	56.7 (36.0, 75.3)	0.15 (0.02, 1.25)	0.61 (0.09, 4.05)

After stratified by household income level (Table 6, column 5), underdiagnosis rate had declined among the lowest income group in seven of 19 countries among the highest income group in five of 15 countries. For both groups, we observed the smallest decline in Belgium (aOR = 0.43 [0.19, 0.96], aOR = 0.22 [0.06, 0.86], respectively) and the greatest decrease in Slovenia (aOR = 0.18 [0.07, 0.49]; aOR = 0.00 [0.00, 0.04], respectively). Compared to the lowest income group (Table 6, column 6), there was more decline in higher income groups in Belgium (middle income group: aOR = 0.22, 95% CI [0.06, 0.83]), Estonia (middle-high income group: aOR = 0.24 [0.07, 0.83]), Poland (middle-high income group: aOR = 0.13 [0.02, 0.85]), Slovenia (high income group: aOR = 0.02 [0.00, 0.14]), and Sweden (middle income group: aOR = 0.10 [0.02, 0.43]).

**Table 6:** Percentage having potential underdiagnosis of dementia by phase and wealth status. Odds ratios (ORs) and their 95% confidence intervals (CIs) I were estimated from weighted logistic regression models for each wealth group, with underdiagnosis of dementia as the dependent variable and phase (end, vs start [reference]) as the predictor, controlling for age, sex, education attainment, marital status, retirement status, number of children, rurality of residence, living in nurse home, number of chronic conditions, hearing loss, utilization of outpatient care, utilization of inpatient care, health insurance status, and proxy status. ORs and their 95% CIs II were estimated from weighted logistic regression models for all wealth groups, with underdiagnosis of dementia as the dependent variable and interaction between phase (end, vs start [reference]) and wealth as the predictor, controlling for age, sex, education attainment, marital status, retirement status, number of children, rurality of residence, living in nurse home, number of chronic conditions, hearing loss, utilization of outpatient care, utilization of inpatient care, health insurance status, and proxy status. “–” means not applicable or unable to estimated due to lacking of corresponding samples. *** *P* < 0.001; ** *P* < 0.01; * *P* < 0.05;. *P* < 0.1

Country	Wealth status (quintile)	Start phase	End phase	Interaction I Adjusted OR for trend (95% CI)	Interaction II Adjusted OR for trend (95% CI)
Austria	Lowest (Q1)	75.6 (65.3, 83.7)	74.2 (57.5, 86.0)	0.91 (0.36, 2.30)	Reference
	Q2	74.5 (62.7, 83.6)	48.2 (25.4, 71.7)	0.31 (0.10, 0.94)*	0.34 (0.08, 1.47)
	Q3	77.4 (64.2, 86.8)	42.5 (18.5, 70.7)	0.18 (0.05, 0.70)*	0.23 (0.05, 1.09)
	Q4	76.9 (64.1, 86.1)	–	–	–
	Highest (Q5)	84.3 (71.0, 92.1)	65.2 (36.4, 85.9)	0.15 (0.02, 0.94)*	0.29 (0.06, 1.39)
Belgium	Lowest (Q1)	81.4 (72.3, 88.1)	67.6 (53.6, 79.1)	0.43 (0.19, 0.96)*	Reference
	Q2	80.7 (71.2, 87.6)	67.3 (46.7, 82.9)	0.50 (0.20, 1.26)	1.02 (0.32, 3.27)
	Q3	88.7 (78.7, 94.4)	45.2 (26.1, 65.8)	0.11 (0.03, 0.35)***	0.22 (0.06, 0.83)*
	Q4	89.3 (78.6, 95.0)	46.3 (19.4, 75.6)	0.09 (0.02, 0.39)**	0.22 (0.05, 1.03)
	Highest (Q5)	90.2 (82.0, 94.9)	68.5 (37.3, 88.8)	0.22 (0.06, 0.86)*	0.48 (0.10, 2.23)
Croatia	Lowest (Q1)	93.0 (83.2, 97.3)	87.0 (67.2, 95.6)	0.43 (0.09, 2.07)	Reference
	Q2	83.2 (70.5, 91.1)	92.3 (74.9, 98.0)	2.37 (0.53, 10.49)	5.18 (0.66, 40.81)
	Q3	70.5 (45.9, 87.1)	69.9 (32.5, 91.8)	0.88 (0.15, 5.35)	2.00 (0.22, 18.10)
	Q4	72.6 (44.0, 89.9)	100.0 (100.0, 100.0)	5.72 (0.91, 35.80)	8.08 (0.66, 99.49)
	Highest (Q5)	76.1 (41.7, 93.4)	58.2 (23.0, 86.7)	0.46 (0.05, 4.56)	0.90 (0.09, 9.25)
Czech Republic	Lowest (Q1)	85.2 (75.2, 91.6)	67.0 (48.2, 81.5)	0.35 (0.12, 0.98)*	Reference
	Q2	89.4 (78.8, 95.1)	67.6 (49.4, 81.7)	0.20 (0.07, 0.58)**	0.70 (0.17, 2.88)
	Q3	83.4 (71.9, 90.8)	37.8 (18.3, 62.2)	0.09 (0.03, 0.29)***	0.37 (0.09, 1.59)
	Q4	84.7 (74.7, 91.2)	52.1 (30.2, 73.3)	0.15 (0.04, 0.52)**	0.55 (0.14, 2.18)
	Highest (Q5)	86.7 (73.5, 93.9)	33.0 (12.7, 62.4)	0.07 (0.02, 0.30)***	0.22 (0.04, 1.19)
Denmark	Lowest (Q1)	72.8 (58.6, 83.5)	78.5 (62.4, 88.9)	1.26 (0.45, 3.47)	Reference
	Q2	86.5 (72.2, 94.0)	68.7 (42.6, 86.6)	0.36 (0.08, 1.52)	0.24 (0.04, 1.31)
	Q3	89.7 (64.2, 97.7)	–	–	–
	Q4	94.9 (66.8, 99.4)	–	–	–
	Highest (Q5)	–	–	–	–
Estonia	Lowest (Q1)	87.9 (81.6, 92.3)	79.4 (68.9, 87.0)	0.51 (0.23, 1.14)	Reference
	Q2	91.5 (84.9, 95.4)	72.8 (59.0, 83.2)	0.24 (0.09, 0.64)**	0.52 (0.16, 1.66)
	Q3	91.1 (84.6, 95.0)	80.6 (66.0, 89.9)	0.34 (0.12, 0.96)*	0.96 (0.28, 3.27)
	Q4	94.5 (88.3, 97.5)	70.0 (55.7, 81.2)	0.11 (0.03, 0.34)***	0.24 (0.07, 0.83)*
	Highest (Q5)	78.7 (65.2, 88.0)	68.2 (37.8, 88.3)	0.30 (0.07, 1.26)	0.78 (0.17, 3.50)
France	Lowest (Q1)	90.9 (85.5, 94.4)	80.1 (69.8, 87.5)	0.38 (0.17, 0.84)*	Reference
	Q2	81.8 (74.5, 87.3)	76.8 (63.1, 86.5)	0.74 (0.34, 1.60)	1.90 (0.64, 5.66)
	Q3	85.3 (76.9, 91.0)	77.3 (56.0, 90.1)	0.64 (0.22, 1.91)	1.63 (0.43, 6.16)
	Q4	90.3 (81.5, 95.2)	83.4 (63.9, 93.4)	0.53 (0.15, 1.94)	1.49 (0.37, 6.05)
	Highest (Q5)	88.1 (77.5, 94.1)	–	–	–
Germany	Lowest (Q1)	78.6 (57.6, 90.9)	52.0 (35.8, 67.7)	0.27 (0.08, 0.94)*	Reference
	Q2	67.9 (45.1, 84.4)	49.2 (30.1, 68.6)	0.44 (0.13, 1.47)	1.61 (0.30, 8.60)
	Q3	82.6 (59.2, 93.9)	46.5 (25.6, 68.6)	0.18 (0.04, 0.81)*	0.66 (0.11, 4.10)
	Q4	79.3 (54.1, 92.5)	32.2 (7.5, 73.4)	0.13 (0.02, 0.95)*	0.46 (0.05, 4.16)
	Highest (Q5)	80.8 (53.2, 93.9)	36.6 (10.5, 74.1)	0.17 (0.03, 1.09)	0.53 (0.06, 4.65)
Greece	Lowest (Q1)	82.3 (75.7, 87.5)	78.5 (67.8, 86.3)	0.83 (0.43, 1.61)	Reference
	Q2	84.7 (77.2, 90.1)	68.3 (56.1, 78.5)	0.39 (0.19, 0.76)**	0.49 (0.18, 1.30)
	Q3	77.3 (67.3, 85.0)	70.6 (55.6, 82.1)	0.70 (0.31, 1.55)	0.88 (0.31, 2.51)
	Q4	78.4 (66.7, 86.7)	66.5 (50.9, 79.2)	0.52 (0.23, 1.20)	0.70 (0.25, 1.96)
	Highest (Q5)	83.7 (68.5, 92.4)	57.1 (36.7, 75.3)	0.22 (0.07, 0.70)*	0.32 (0.10, 1.09)
Hungary	Lowest (Q1)	75.7 (55.1, 88.7)	63.6 (23.0, 91.1)	0.59 (0.09, 4.12)	Reference
	Q2	89.0 (65.9, 97.1)	73.1 (29.8, 94.6)	0.54 (0.05, 5.38)	0.64 (0.04, 9.44)
	Q3	78.1 (48.3, 93.1)	–	–	–
	Q4	77.4 (60.0, 88.6)	–	–	–
	Highest (Q5)	30.5 (6.0, 75.1)	–	–	–
Italy	Lowest (Q1)	90.4 (83.0, 94.8)	77.4 (59.2, 89.0)	0.33 (0.12, 0.92)*	Reference
	Q2	93.2 (86.7, 96.7)	85.0 (74.4, 91.7)	0.40 (0.15, 1.05)	1.14 (0.28, 4.74)
	Q3	88.8 (79.0, 94.4)	79.9 (66.1, 89.0)	0.46 (0.18, 1.20)	1.40 (0.34, 5.73)
	Q4	92.2 (80.9, 97.1)	70.1 (52.7, 83.2)	0.14 (0.04, 0.46)**	0.55 (0.11, 2.80)
	Highest (Q5)	85.4 (72.1, 93.0)	79.9 (48.9, 94.3)	0.63 (0.11, 3.68)	1.86 (0.29, 12.08)
Luxembourg	Lowest (Q1)	79.6 (56.6, 92.1)	70.3 (42.5, 88.4)	0.54 (0.11, 2.74)	Reference
	Q2	82.4 (61.4, 93.2)	60.5 (33.1, 82.6)	0.33 (0.05, 1.98)	0.59 (0.07, 5.01)
	Q3	81.6 (59.9, 92.9)	31.1 (9.4, 66.2)	0.10 (0.02, 0.54)**	0.18 (0.02, 1.78)
	Q4	74.2 (51.0, 88.9)	–	–	–
	Highest (Q5)	91.0 (46.3, 99.2)	47.1 (18.5, 77.8)	0.02 (0.00, 1.07)	0.22 (0.01, 4.43)
Netherlands	Lowest (Q1)	78.0 (63.8, 87.7)	59.9 (36.8, 79.3)	0.33 (0.10, 1.12)	Reference
	Q2	79.4 (61.7, 90.2)	67.5 (44.6, 84.3)	0.63 (0.18, 2.24)	1.33 (0.25, 7.11)
	Q3	91.2 (78.1, 96.8)	58.1 (29.0, 82.5)	0.14 (0.03, 0.70)*	0.35 (0.05, 2.30)
	Q4	86.5 (60.2, 96.4)	28.1 (6.5, 68.9)	0.10 (0.01, 0.97)*	0.18 (0.02, 1.78)
	Highest (Q5)	84.7 (57.2, 95.8)	46.0 (14.3, 81.3)	0.15 (0.02, 1.01)	0.36 (0.04, 3.30)
Poland	Lowest (Q1)	80.0 (66.9, 88.7)	74.9 (64.4, 83.1)	0.62 (0.26, 1.49)	Reference
	Q2	91.5 (82.3, 96.1)	79.3 (62.9, 89.6)	0.40 (0.14, 1.11)	0.48 (0.12, 1.91)
	Q3	83.7 (71.4, 91.4)	79.4 (63.8, 89.3)	0.62 (0.21, 1.86)	1.09 (0.29, 4.05)
	Q4	100.0 (100.0, 100.0)	69.2 (46.9, 85.1)	0.09 (0.02, 0.53)**	0.13 (0.02, 0.85)*
	Highest (Q5)	89.9 (74.4, 96.5)	57.0 (26.1, 83.3)	0.20 (0.03, 1.14)	0.24 (0.04, 1.42)
Portugal	Lowest (Q1)	80.7 (44.7, 95.6)	84.2 (66.0, 93.6)	1.24 (0.23, 6.80)	Reference
	Q2	94.7 (86.2, 98.1)	89.8 (77.4, 95.8)	0.29 (0.07, 1.22)	0.27 (0.03, 2.81)
	Q3	87.1 (66.0, 95.9)	81.6 (58.6, 93.3)	0.59 (0.11, 3.21)	0.47 (0.04, 5.28)
	Q4	75.7 (50.7, 90.5)	96.0 (84.3, 99.1)	7.00 (1.17, 41.68)*	6.97 (0.53, 91.25)
	Highest (Q5)	89.2 (55.7, 98.2)	60.5 (23.3, 88.5)	0.07 (0.00, 1.26)	0.12 (0.01, 2.29)
Slovenia	Lowest (Q1)	92.4 (82.9, 96.8)	68.8 (58.1, 77.8)	0.18 (0.07, 0.49)***	Reference
	Q2	87.0 (72.7, 94.4)	75.1 (61.0, 85.3)	0.39 (0.12, 1.26)	2.28 (0.51, 10.15)
	Q3	90.7 (79.0, 96.2)	61.4 (43.5, 76.6)	0.14 (0.04, 0.48)**	0.83 (0.18, 3.83)
	Q4	81.2 (62.4, 91.8)	36.1 (19.9, 56.2)	0.12 (0.04, 0.43)**	0.66 (0.13, 3.26)
	Highest (Q5)	98.6 (94.4, 99.7)	22.3 (8.6, 46.7)	0.00 (0.00, 0.04)***	0.02 (0.00, 0.14)***
Spain	Lowest (Q1)	89.4 (85.1, 92.6)	78.7 (69.2, 85.9)	0.41 (0.22, 0.75)**	Reference
	Q2	92.0 (87.5, 94.9)	82.6 (70.6, 90.4)	0.39 (0.19, 0.83)*	0.96 (0.36, 2.55)
	Q3	92.2 (87.6, 95.2)	67.2 (50.8, 80.3)	0.17 (0.07, 0.39)***	0.39 (0.14, 1.11)
	Q4	92.4 (87.1, 95.6)	85.0 (67.5, 93.9)	0.45 (0.15, 1.41)	1.12 (0.31, 4.05)
	Highest (Q5)	89.0 (82.1, 93.5)	75.7 (48.4, 91.2)	0.43 (0.13, 1.40)	0.87 (0.21, 3.53)
Sweden	Lowest (Q1)	50.9 (34.9, 66.8)	61.4 (46.5, 74.4)	1.61 (0.65, 3.97)	Reference
	Q2	59.2 (40.1, 75.8)	38.9 (19.2, 63.0)	0.44 (0.12, 1.72)	0.28 (0.06, 1.23)
	Q3	82.9 (66.0, 92.4)	44.6 (26.4, 64.4)	0.16 (0.05, 0.54)**	0.10 (0.02, 0.43)**
	Q4	74.4 (43.3, 91.7)	61.2 (31.7, 84.3)	0.52 (0.07, 3.74)	0.39 (0.06, 2.48)
	Highest (Q5)	–	–	–	–
Switzerland	Lowest (Q1)	77.9 (63.0, 87.9)	64.1 (44.8, 79.7)	0.54 (0.18, 1.62)	Reference
	Q2	72.3 (52.5, 86.1)	73.8 (50.5, 88.6)	1.08 (0.28, 4.15)	1.94 (0.37, 10.18)
	Q3	85.2 (60.3, 95.6)	–	–	–
	Q4	83.2 (54.0, 95.5)	–	–	–
	Highest (Q5)	–	–	–	–

## Discussion

### Principal findings

As we hypothesized, the underdiagnosis of probable dementia experienced an overall decrease from 2011–2015 to 2015–2019, but the magnitude of change varied across the 19 European countries included in this study. At a national level, the risk of underdiagnosis was negatively associated with the availability of specialized psychiatric resources such as psychiatrists and psychiatric hospital beds. However, it was positively associated with the supply of general or advanced healthcare resources like general practitioners, at-home long-term care workers, and PET scanners. On an individual level, individuals who were older, more educated, living in a nursing home, with multimorbidity, and proxy-reported had lower risk of underdiagnosis. In contrast, those who were married, free of chronic conditions, and using outpatient services had higher risk. Progress in underdiagnosis reduction was positively associated with investments in psychiatric care, home care services, and PET imaging. Such progress was also modified by factors including age, educational attainment, retirement status, nursing home residency, multimorbidity, and healthcare utilization. Country-specific analyses showed consistent decline in underdiagnosis rate in most countries across different age groups, sex, and income levels, while a few countries observed less reduction or even increasing rates among individuals aged 80 or above. Overall, there was more significant diagnosis improvement in males and individuals with higher income.

### Rates and trends of underdiagnosis across countries

The variation in dementia underdiagnosis rates across European countries may be due to the differences in healthcare systems, national strategies, cultural attitudes, and resource allocation. Countries with low underdiagnosis rates, like Hungary (42.6%) and Germany (47.8%), have well-developed healthcare systems and specific initiatives for dementia diagnosis [21–23]. In contrast, countries with high underdiagnosis rates, such as Croatia (86.6%) and Portugal (85.2%), may face challenges related to limited resources, fragmented healthcare systems, and lack of prioritization of dementia [21]. Cultural factors, such as stigma and regional disparities, may also contribute to higher underdiagnosis rates in France (78.9%) and Italy (80.1%) [35, 36]. We found that higher with availability of psychiatrists and psychiatric hospital beds was associated with reduced underdiagnosis risk, supporting the effectiveness of specialized mental health professionals and facilities in improving dementia diagnosis [37]. This finding aligns with the recommendations outlined in the National Institute for Health and Care Excellence (NICE) guideline in the UK, which emphasizes the necessity of dementia diagnoses being conducted by qualified specialists [38]. Surprisingly, we found that higher supply of general or advanced healthcare resources (more GPs and formal LTC workers at home), which are typically expected to increase clinical diagnosis capacity, was associated with higher risk of probable dementia underdiagnosis in our study. This suggests that simply increasing these professionals may not necessarily improve diagnosis rates due to lack of dementia-specific training, time constraints, and insufficient resources [37, 39]. The contrasting contribution of general and specialized healthcare resources highlight the importance in dementia-specific expertise in improving diagnosis ability.

Our findings revealed a significant decrease in the rate of underdiagnosis of probable dementia in 15 out of 19 European countries between 2011–2015 and 2015–2019, with varying magnitudes of decline across countries. This declining trends may be attributed to implantation of national dementia strategies focusing on early diagnosis, awareness, and care in these countries, while the variation in these trends may be partially explained by the stage and effectiveness of these strategies [24]. For example, Switzerland launched its National Dementia Strategy 2014–2019, which aimed to improve early detection and diagnosis [40]. Differences in [40]. Progress in improving diagnosis may also be affected by differences in availability and accessibility of specialist services, such as memory clinics and geriatric psychiatry [41]. Other contributing factors could include the implementation and effectiveness of initiatives including public awareness campaigns, efforts to reduce stigma, adoption of new diagnostic tools, implementation of clinical guidelines, and enhancing the knowledge and skills of healthcare professionals in recognizing and diagnosing dementia [23, 41–43].

### Individual-level contributing factors

Our results identified several individual-level contributors to the risk of dementia underdiagnosis and the progress in reducing this underdiagnosis from 2011–2015 to 2015–2019. Older age, higher education, living in a nursing home, and multimorbidity were associated with a lower risk of underdiagnosis, possibly related to health awareness, recognition of cognitive changes, and access to healthcare services [13, 14, 44, 45]. Older individuals and those with higher education may be more proactive in seeking medical attention for cognitive concerns, while nursing home residents may receive more frequent monitoring and assessment by healthcare professionals [44]. Being married was associated with higher risk of underdiagnosis, potentially due to reliance on spousal support. Surprisingly, we found individuals without chronic conditions had higher risk of underdiagnosis. This is possibly because they have lower utilization of healthcare [13, 44]. This finding highlights a potential particularly vulnerable population for targeted dementia screening interventions—those who remain disconnected from regular healthcare due to absence of other health conditions requiring medical attention. Use of outpatient services was associated with a higher risk of underdiagnosis but inpatient care showed no significant association. This suggests potential gaps in the detection and diagnosis of dementia in outpatient settings [37, 46]. The type, quality, or continuity of outpatient care may influence the likelihood of dementia diagnosis [43, 47]. There was grater decline in the risk of underdiagnosed probable dementia among individuals who were more educated, retired, utilizing outpatient care, and proxy-reported. This indicates the effectiveness of efforts to improve diagnosis for these subgroups. We identified slower progress of improving diagnosis among individuals aged 80–90 years, living in nursing home, without chronic diseases, and using inpatient care. These results suggest potential gaps and challenges in these subgroups, for example, the complexity of diagnosing dementia in the presence of multiple comorbidities and functional impairments [9, 14]. More targeted strategies are needed to identify and diagnose dementia in these populations.

### Country-level contributing factors

While greater availability of medical equipment related to dementia diagnosis is generally expected to reduce underdiagnosis of dementia, our findings suggest a more complex relationship. In our study, country-level availability of CT and MRI scanners was not significantly associated with the risk of underdiagnosing dementia; availability of PET scanners was positively associated with the risk. This discrepancy may be explained by several factors. First, PET scans have shown favorable sensitivity and specificity in diagnosis of dementia [48], particularly Alzheimer’s disease. Therefore, the number of PET scanners could serve as a proxy indicator of a healthcare system’s capacity to accurately diagnose dementia [48]. However, interpreting PET scans for dementia diagnosis requires specific expertise. In the case of limited qualified professionals, the availability of PET scanners may not necessarily translate to accurate diagnosis [49]. Moreover, the high-cost PET scans may limit access for certain population subgroups, thereby contributing to underdiagnosis in these groups [50]. Lastly, PET scans are more often used in patients with advanced or complex presentations of dementia, which may result in missed diagnosis in early-stage or atypical cases.

Our analyses identified several factors associated with progress in reducing dementia underdiagnosis between 2011–2015 and 2015–2019. These factors included higher numbers of psychiatrists and formal LTC workers at home or in institutions and higher availability of PET scanners. The increase in number of psychiatrists, particularly those with geriatric psychiatry expertise, likely facilitated improved access to specialized diagnostic services, more accurate and timely diagnoses, and better coordination of care [37]. The positive contribution of increased formal LTC workers indicates the importance of a skilled workforce in early detection and management of dementia. The LTC workers can observe and report changes in cognitive function and daily living skills of individuals, which can prompt further assessment and diagnosis [51]. PET scanner availability was positively associated with reduction in underdiagnosis. This suggests that access to advanced neuroimaging techniques may improve diagnostic accuracy and efficiency, particularly in atypical presentations or unclear clinical findings [48].

In our study, higher availability of formal LTC workers at home and PET scanners was associated with higher risk of underdiagnosis but greater reduction in underdiagnosis over time. These seemingly contradictory findings can be explained by several factors. First, there may be a threshold effect on benefits of LTC workers and PET scanners on dementia, where meaningful improvements in diagnosis are only observed at a certain level of availability or utilization. Improvements in the training and education of LTC workers, advances in PET imaging techniques, and development of standardized protocols for PET interpretation over the study period may have contributed to the observed progress in reducing underdiagnosis, despite the initial risks [42, 43, 49, 52]. Moreover, broader healthcare system factors, such as referral procedures, access to specialist services, and reimbursement policies, likely influenced how the availability of LTC worker and PET scanner translate into improved diagnostic outcomes over time [7, 37, 39, 46]. Further research is needed to examine the temporal changes in the utilization of these factors and their complex interactions with other elements of the healthcare system. A more comprehensive understanding of these relationships will help to inform future strategies to reduce underdiagnosis.

### Potential disparities in diagnosis

The subgroup analysis identified consistent progress in reducing underdiagnosis across age groups in most countries. This reflects consistent efforts to improve dementia diagnosis across the age spectrum. However, we noted non-significant reduction in underdiagnosis among people aged 80–90 in Germany and even increased underdiagnosis among those aged 90 and over in France and Sweden. These findings indicate potential challenges in diagnosing dementia in the oldest age group, possibly due to comorbidities, frailty, functional impairments, and the perception that cognitive decline is a normal part of aging [9, 14]. The comparable progress between males and females and across different income groups suggests equitable efforts to improve dementia diagnosis. However, the greater progress among males in Austria, Belgium, Luxembourg, and the Netherlands may reflect sex-specific differences in health-seeking behavior and access to healthcare services, as well as targeted initiatives to raise awareness and encourage help-seeking among men [8]. The higher income groups showed greater diagnosis progress in Belgium, Estonia, Poland, Slovenia, and Sweden. This suggests potential disparities in access to and utilization of diagnostic services, possibly due to better access to healthcare resources, greater health literacy, and increased awareness of dementia among individuals from higher socioeconomic backgrounds [8].

### Implications

The identified risk factors of underdiagnosis and progress in reducing underdiagnosis can inform targeted interventions to bridge the gap in dementia diagnosis and care. These interventions include dementia-specific education for GPs and LTC workers, integrating cognitive assessment tools, establishing referral pathways, and implementing collaborative care models [42, 43]. A multifaceted approach is needed to improve dementia diagnosis, which involves training and recruiting specialized mental health professionals, developing dedicated facilities for dementia care, and adopting diagnostic technologies, while taking into account each country’s specific healthcare system, policies, and cultural context. The subgroup analyses revealed potential disparities in diagnostic improvement across age, gender, and socioeconomic status in some countries. Further research is needed to understand the underlying factors contributing to these disparities and to develop targeted interventions to ensure equitable access to dementia diagnosis and care.

### Strengths and limitations

To our knowledge, this is the first study to assess the underdiagnosis of dementia across 19 European countries representing diverse healthcare systems, policies, and cultural contexts. The repeated cross-sectional representative data allows for the assessment of temporal trends and progress in reducing underdiagnosis. This study used data of SHARE, which explicitly used standardized methods across participating countries to support cross-national comparisons. Moreover, the study examined a comprehensive set of individual-level and country-level predictors, including sociodemographic characteristics, healthcare utilization, and national healthcare resources. This design provides a better understanding of the complex interplay of factors influencing dementia diagnosis. The subgroup analyses by age, sex, and income status further strengthened the findings by identifying disparities across key demographic dimensions.

The present study has several limitations. First, the definition of probable dementia relies on a limited set of cognitive tests. However, previous validation studies have demonstrated a 78% consistency in dementia diagnoses when using these tests compared to comprehensive The Aging, Demographics, and Memory Study (ADAMS) clinical assessments [53]. Second, the self-reported data in this study may introduce recall bias or underreporting that could influence our findings. This applies not only to individual-level predictors (like outpatient or inpatient visits) but importantly also to our primary outcome measure, which relies on self-reported diagnosis of dementia. Individuals with cognitive impairment may be less likely to accurately recall or report a formal diagnosis, potentially leading to an overestimation of underdiagnosis rates. This limitation is partially mitigated by our inclusion of proxy respondents participants with more severe cognitive impairment, though recall bias may still affect proxy responses. Third, individuals’ physical health, such as hearing loss, may affect the results of this study. Hearing loss potentially undermines the accuracy of cognitive tests conducted through telephone interviews in SHARE [54, 55]. Although we controlled for hearing loss in individual-level regression, the impact of this limitation cannot be ruled out. Fourth, while the study included diverse European countries, the findings may not be directly applicable to other regions with different healthcare systems, cultural norms, or socioeconomic conditions. Moreover, country-level analyses should be interpreted with caution. While public health expenditure is a common indicator of national-level healthcare resources and accessibility, it may not fully capture costs associated with dementia diagnosis in countries without universal health coverage. Additionally, metrics such as psychiatric bed availability and psychiatrist density may not serve as ideal proxies of dementia diagnosis capacity, as uncomplicated dementia cases are typically diagnosed by neurologists, whereas psychiatrists are more often involved in managing cases with complex neuropsychiatric presentations. However, due to the absence of specific indicators on neurologists and geriatricians in Eurostat data, we were unable to include them in our analysis. This highlights the need for improved data availability, given the significant burden of dementia across the EU. Additionally, it should also be noted that hospital equipment like CT, MRI, and PET scanners is used for diagnosing a wide range of conditions beyond dementia. Therefore, country-level results provide a macro-level perspective that may reflect multiple underlying factors, such as reallocating savings from reducing psychiatric beds to improve mental healthcare in primary or outpatient secondary care settings, or efforts to raise public awareness on dementia. Another limitation is the small sample sizes in certain subgroups, particularly for the oldest age category (≥ 90 years) and the highest income quintile in several countries. The insufficient data in certain groups, especially in country-specific analyses, may affect the precision and reliability of our subgroup estimates, leading to missing estimates or extreme estimates such as odds ratios that are very large or close to zero. The small sample sizes in these analyses may lead to wider confidence intervals, extreme point estimates, and reduced statistical power, which should be considered when interpreting the results. Further, there may be unmeasured confounders, such as lifestyle behaviors or access to specialized dementia services, that could contribute to diagnostic outcomes. Lastly, we did not examine how demographic factors such as sex might moderate the relationships between other individual-level predictors (e.g., marital status) and underdiagnosis risk. For instance, the effect of being married on underdiagnosis risk might differ between males and females, potentially being harmful for one group while beneficial for another. Future research should include a more comprehensive range of factors to better understand the underdiagnosis of dementia, as well as explore the potential interaction effects to provide a more nuanced understanding of the complex interplay between multiple demographic and social factors in determining dementia diagnostic outcomes.

## Conclusions

In European countries, there was an overall reduction in underdiagnosis of probable dementia over time, with varying progress across countries. To improve diagnosis, it is important to increase availability and access to specialized psychiatric resources, promote targeted training, and implement collaborative care models. Interventions should prioritize population groups at higher risk of underdiagnosis, such as older individuals, nursing home residents, and those without chronic diseases. To reduce diagnostic inequalities, equitable efforts are needed to ensure improvements across age, gender, and socioeconomic status.

## Data Availability

The data are publicly available and can be accessed here (https://share-eric.eu/data/).

## Abbreviations

ADAMS: The Aging, Demographics, and Memory Study
aOR: Adjusted odds ratio
CI: Confidence interval
CT: Computed tomography
GP: General practitioner
HRS: Health and Retirement Survey
IADLs: Instrumental activities of daily living
LTC: Long-term care
MRI: Magnetic resonance imaging
NICE: National Institute for Health and Care Excellence
PET: Positron emission tomography
SD: Standard deviation
SHARE: Survey of Health, Ageing, and Retirement in Europe
UK: United Kingdom
US: United States
WHO: World Health Organization
